# *In Vitro* Toxicity of Epigallocatechin Gallate in Rat Liver Mitochondria and Hepatocytes

**DOI:** 10.1155/2015/476180

**Published:** 2015-03-30

**Authors:** Otto Kucera, Vojtech Mezera, Alena Moravcova, Rene Endlicher, Halka Lotkova, Zdenek Drahota, Zuzana Cervinkova

**Affiliations:** ^1^Department of Physiology, Faculty of Medicine in Hradec Kralove, Charles University in Prague, 500 38 Hradec Kralove, Czech Republic; ^2^Department of Anatomy, Faculty of Medicine in Hradec Kralove, Charles University in Prague, 500 38 Hradec Kralove, Czech Republic

## Abstract

Epigallocatechin-3-gallate (EGCG) is the main compound of green tea with well-described antioxidant, anti-inflammatory, and tumor-suppressing properties. However, EGCG at high doses was reported to cause liver injury. In this study, we evaluated the effect of EGCG on primary culture of rat hepatocytes and on rat liver mitochondria in permeabilized hepatocytes. The 24-hour incubation with EGCG in concentrations of 10 *μ*mol/L and higher led to signs of cellular injury and to a decrease in hepatocyte functions. The effect of EGCG on the formation of reactive oxygen species (ROS) was biphasic. While low doses of EGCG decreased ROS production, the highest tested dose induced a significant increase in ROS formation. Furthermore, we observed a decline in mitochondrial membrane potential in cells exposed to EGCG when compared to control cells. In permeabilized hepatocytes, EGCG caused damage of the outer mitochondrial membrane and an uncoupling of oxidative phosphorylation. EGCG in concentrations lower than 10 *μ*mol/L was recognized as safe for hepatocytes *in vitro*.

## 1. Introduction

Epigallocatechin-3-gallate (EGCG) is the most abundant and potent catechin of green tea [1]. Its cytoprotective effect was reported in the liver [2–4] and other organs, for example, kidney [5, 6], heart [7, 8], and lungs [9]. The protection is mediated partly by its direct antioxidant effect [10–12] and partly by the induction of Nrf2-dependent gene expression [5, 10, 13]. This includes activation of superoxide dismutase, catalase, and glutathione S-transferase in the liver [14, 15]. Other mechanisms involved are inhibition of expression of proinflammatory mediators such as tumor necrosis factor *α* (TNF*α*), cyclooxygenase 2, and inducible nitric oxide synthase [16], reduction of nuclear factor *κ*B activity [1, 17, 18], and elevation of CCAAT-enhancer-binding protein-*α* expression [16]. Some of the anti-inflammatory actions of EGCG can be attributed to the 67 kDa laminin receptor signaling [19].

By inhibiting the expression of platelet-derived growth factor (PDGF) receptor-*β* and insulin-like growth factor 1 receptor [20] as well as by suppression of matrix metalloproteinase 2 [21], EGCG was able to prevent the development of liver fibrosis in rats treated with carbon tetrachloride; a similar effect was observed in mice [16, 22]. Its antifibrotic effect can also be explained by the induction of de novo glutathione synthesis and transforming growth factor *β* signaling interruption [23]. Green tea polyphenols were found to protect from acetaminophen- [24] and D-galactosamine-induced hepatotoxicity [25]. EGCG and/or green tea attenuated high-fat diet-induced nonalcoholic steatohepatitis [4, 26] and displayed a similar effect in a model of steatohepatitis in ob/ob mice [27, 28]. Recently, an ability of EGCG to induce hepatic autophagy was suggested [26]. In addition, green tea extract [29–31] and EGCG [32] displayed protective effects against ethanol-induced hepatotoxicity.

Ischemia/reperfusion injury was attenuated in both normal [18] and steatotic liver [28]. The effect of EGCG on liver regeneration after partial hepatectomy seems to be dependent on dose and way of administration: Saito et al. described an improvement of liver regeneration in rats receiving water with 0.5% green tea extract including EGCG [33], while we observed a rather inhibitory effect of EGCG on liver regeneration when administered in repeated doses of 50 mg/kg intraperitoneally [34].

Furthermore, EGCG was able to restore the activity of glutathione peroxidase and glutathione levels and to inhibit the production of hydrogen peroxide and nitric oxide in human skin [35]. EGCG may reduce the risk of cardiovascular diseases via interference with PDGF signaling and inhibition of vascular smooth muscle mitogenesis [36], by activation of endothelial nitric oxide synthase and via a phosphatidylinositol 3-kinase/Akt-dependent pathway [37, 38]. In addition, EGCG decreased hepatic VLDL secretion and enhanced biliary secretion of cholesterol [39].

On the other hand, overdose with EGCG and/or green tea extract can lead to liver injury [40–43]. The injury can be explained by the prooxidant effect of high doses of EGCG [11, 41, 44] as well as by a decline in levels of reduced glutathione [43, 45] and mitochondrial membrane potential collapse [43]. This eventually leads to hepatic necrosis and neutrophil infiltration [46].

There is a conflicting evidence of the influence of EGCG on mitochondria: some authors reported a protective effect of EGCG [47–50] and decrease of reactive oxygen species formation in the mitochondria [12]. Other groups found an inhibitory effect of EGCG on the activity of oxidative phosphorylation enzymes [51, 52] and on respiration of mitochondria under swelling state [53].

Therefore, we decided to test the effect of various doses of EGCG on primary rat hepatocyte culture and on liver mitochondria in permeabilized hepatocytes.

## 2. Methods

### 2.1. Chemicals

All chemicals were, unless otherwise stated, of analytical grade and obtained from Sigma-Aldrich (Madison, WI, USA). The Rat TNF*α* Platinum ELISA kit was obtained from eBioscience (San Diego, CA, USA), Rat Albumin ELISA Quantitation kit was from Bethyl Laboratories (Montgomery, TX, USA). Kit for measurement of lactate dehydrogenase activity was obtained from DiaSys (Holzheim, Germany), substrate for WST-1 test was obtained from Roche (Penzberg, Germany). Fluorescence probe dichlorodihydrofluorescein diacetate (DCFDA) was obtained from Life Technologies (Carlsbad, CA, USA).

### 2.2. Animals

Male Wistar rats (268 ± 45 g) were obtained from Velaz (Lysá nad Labem, Czech Republic) and fed a standard laboratory diet* ad libitum*. The animals were housed at 23 ± 1°C, 55% ± 10% humidity, with air exchange 12–14 times/h, and a 12 h (light)/12 h (dark) cycle. All experimental protocols were approved by the Institutional Animal Use and Care Committee of the Faculty of Medicine in Hradec Kralove. All invasive procedures were performed under general anesthesia.

### 2.3. Hepatocyte Isolation

The primary hepatocytes were isolated under ether anesthesia as described by Berry et al. [54] with small modifications [55]. Viability of isolated cells, determined by a trypan blue exclusion test, was higher than 90% in all experiments. After isolation, the cells were inoculated on collagen-coated 6-well (1 × 10^6^ cells/well in 1 mL), 24-well (3 × 10^5^ cells/well in 0.3 mL), and 96-well (3.3 × 10^4^ cells/well in 0.1 mL) culture plates (NUNC, Thermo Scientific, Waltham, MA, USA) and placed in a humidified atmosphere (37°C, 5% CO_2_). The cells were allowed to attach for 2 hours in the presence of a Williams E medium supplemented with 6% fetal bovine serum (Merck-Millipore, Berlin, Germany), glutamine (2 mmol/L), penicillin (100 IU/mL), streptomycin (10 mg/mL), insulin (0.08 IU/mL), dexamethasone (0.12 *μ*g/mL), and glucagon (0.008 *μ*g/mL). Afterwards, medium was replaced by a medium without fetal bovine serum and the cells were exposed to various concentrations of EGCG ranging from 5 to 100 *μ*mol/L.

### 2.4. Microscopic Evaluation

All images were taken using Olympus IX51 microscope with E-600 Digital Camera (Olympus Imaging Corp., Tokyo, Japan) and Quick Photo Camera 3.0 software (Promicra, Prague, Czech Republic). Scale bars (50 *μ*m) were added using the Quick Photo Camera software; no other image editing was performed. We used 2 types of visualization: phase contrast (40x objective) and JC-1 staining (40x objective) as described previously [55].

### 2.5. Markers of Cell Viability and Function

As a marker of cell viability, we performed the WST-1 test according to manufacturer's instructions. Briefly, after removing the medium, cells were exposed to WST-1 reagent diluted in fresh Williams E medium, 1 : 10. The absorbance at 440 nm was measured at time 0 and 75 min and the difference was calculated.

As a marker of cell membrane damage, we measured the intra- and extracellular activity of lactate dehydrogenase (LDH) and calculated LDH leakage as a percentage of the activity in the medium divided by total activity [56].

The production of albumin in the medium was measured using ELISA method.

### 2.6. Activity of Caspases 3, 8, and 9

The activity of caspases 3, 8, and 9 was measured in cell lysate (Cell Lysis Buffer, Cell Signaling Technology, Danvers, MA, USA) after addition of specific substrates Ac-DEVD-AMC, Ac-LETD-AFC, and Ac-LEHD-AMC, respectively (Enzo Life Sciences, Farmingdale, NY). The activities were measured in a fluorescent mode using a TECAN Infinite M200 spectrofluorometer (Tecan Group AG, Männedorf, Switzerland). The excitation and emission wavelengths were 350 and 460 nm, for caspase 3 and caspase 9, and 400 and 505 nm for caspase 8.

### 2.7. DAPI Staining

To further assess cell apoptosis, hepatocytes on 6-well plates were cultivated with EGCG at concentrations of 0, 10, 30, and 100 *μ*mol/L for up to 24 hours. In 4-hour intervals, hepatocytes were stained with DAPI (2-(4-amidinophenyl)-6-indolecarbamidine dihydrochloride, 2 *μ*g/mL, 15 min) and count of apoptotic cells (chromatin condensation and fragmentation) per visual field (40x) was examined using a fluorescence microscopy. At least 10 fields from different area of well plate were subjected to visual score for each sample.

### 2.8. Cytokines Production

The concentrations of proinflammatory cytokine TNF*α* after 24 h incubation with EGCG were measured in the medium using ELISA kits.

### 2.9. Reactive Oxygen Species Formation

We evaluated the formation of reactive oxygen species using a fluorescent probe DCFDA as described previously [56].

We estimated the rate of lipid peroxidation by measurement of levels of malondialdehyde in cell lysate (Cell Lysis Buffer) by the TBARS (thiobarbituric acid-reactive substances) method [57].

### 2.10. Mitochondrial Respiration Measurement

Oxygen consumption was measured by a high-resolution respirometry using Oxygraph 2k (Oroboros Instruments, Innsbruck, Austria) as described previously [55, 58]. First, the suspension of hepatocytes (125,000/mL) was permeabilized with digitonin (10 *μ*g/mL) in K^+^-medium and then the cells were exposed to various concentrations of EGCG from 10 to 200 *μ*mol/L for 10 minutes. Respiration rates were measured at baseline and after addition of substrates of complex I (glutamate and malate, state 4), followed by addition of ADP (state 3), cytochrome c (for testing of the outer mitochondrial membrane integrity), rotenone (an inhibitor of complex I), and succinate (a substrate of complex II). The consumption of oxygen was measured in pmol O_2_ per second and million of cells. Respiratory control ratio (RCR) of complex I (a ratio of consumption rate in state 3 to state 4) was calculated. Data were analyzed by Oroboros DatLab 5.1 (Oroboros Instruments).

### 2.11. Statistical Evaluation

The experiments were performed at least three times; representative results are shown. Values are depicted as mean ± SD; *P* < 0.05 was set as the border for statistical significance. Statistical evaluation was performed using GraphPad Prism software (La Jolla, CA, USA). Data were first tested for normality by means of Kolmogorov-Smirnoff test. In the case of Gaussian distribution, data were further analyzed by parametric ANOVA and Dunnett's posttest. In the case of non-Gaussian distribution, data were analyzed by a nonparametric Kruskal Wallis test and Dunn's posttest.

## 3. Results

### 3.1. Cell Viability and Function

**Figure 1:**
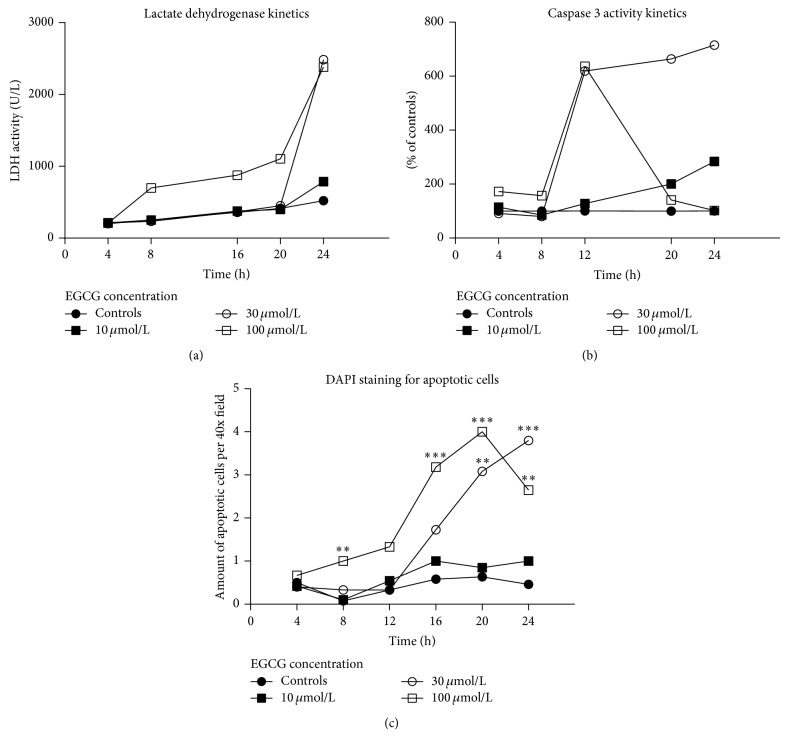
Time-course of cell death. (a) Kinetics of lactate dehydrogenase activity in the culture medium. (b) Kinetics of caspase 3 activity in cell lysate. (c) Kinetics of average number of apoptotic cells per 40x field using DAPI staining (∗∗, ∗∗∗ versus medium-treated controls at corresponding time points; *P* < 0.01, 0.001, resp.).

The LDH leakage was significantly increased in cells exposed to 10 *μ*M EGCG (*P* < 0.001) and higher for 24 h when compared to controls (Table 1). Concentration of 100 *μ*mol/L led to a distinct increase of extracellular LDH already after 8 hours (Figure 1(a)).

**Figure 2:**
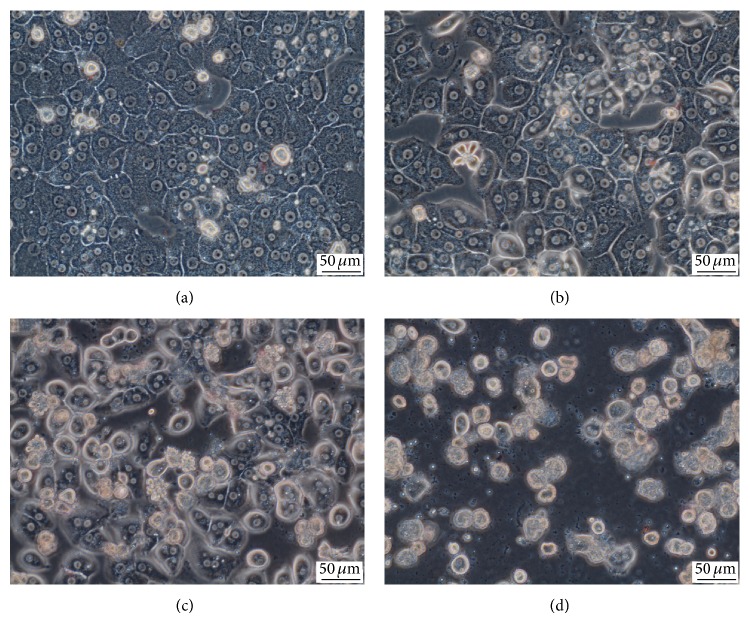
Phase contrast microscopy. (a) Control medium-treated hepatocytes; hepatocytes treated with EGCG at concentration of (b) 10 *μ*mol/L, (c) 30 *μ*mol/L, and (d) 100 *μ*mol/L. Objective magnification 40x.

Concurrently, there was a dose-dependent decline in the activity of cellular dehydrogenases; this decline was significant in cells exposed to 10 *μ*mol/L EGCG (*P* < 0.05) and higher (Table 1). Furthermore, we observed signs of damage in phase contrast microscopy, specifically loss of nuclear visibility, granulation of cytoplasm, and high content of cellular debris (Figures 2(a)–2(d)). We observed a dose-dependent decline in albumin production which was significant even from concentrations of 5 *μ*mol/L (*P* < 0.01) (Table 1). Taken together, these results suggest hepatocellular damage from the concentration of 10 *μ*mol/L EGCG.

### 3.2. Apoptosis Assessment

The time-course of caspase 3 activity in cell lysate from 96-well plates is depicted in Figure 1(b). A steep increase in caspase 3 activity was observed between 8 and 12 hours of incubation with 30 and 100 *μ*M EGCG. After 20 and 24 hours of exposure to 100 *μ*M EGCG, there was a return of caspase 3 activity to control values.

In 24-well plates, we evaluated the activities of caspases 3, 8, and 9 in cell lysate after 24 hours. The activity of caspase 3 was significantly elevated from EGCG concentration of 10 *μ*mol/L (*P* < 0.001) and reached maximum activity at 50 *μ*M EGCG. Caspase 8 was also activated by EGCG treatment (*P* < 0.001 for 30 *μ*mol/L and higher). The activation of caspase 9 activity was less pronounced than the activation of the former ones but was still significant for 30 *μ*mol/L EGCG and higher (*P* < 0.05) (Table 1).

The time-course of DAPI-positive apoptotic hepatocytes on 6-well plates is depicted in Figure 1(c). A steep increase in the number of apoptotic cells was slightly delayed after the rise of caspase 3 activity and was noticed between 12 and 16 hours of cultivation with 30 and 100 *μ*M EGCG.

### 3.3. Cytokines Production

EGCG displayed a biphasic effect on TNF*α* concentrations in culture medium. The lowest tested dose of EGCG (5 *μ*mol/L) showed a nonsignificant 4-fold decrease in TNF*α* level in comparison to control cells. At 15 to 50 *μ*mol/L of EGCG, the levels were higher than in control cells (*P* < 0.05 for 30 *μ*mol/L). At the highest concentration of EGCG (100 *μ*mol/L), the levels of TNF*α* were similar to those in control cells (Table 1).

### 3.4. Reactive Oxygen Species

The formation of reactive oxygen species (ROS) as measured by DCFDA displayed a decline after low doses of EGCG significant for concentrations 10 and 15 *μ*mol/L (*P* < 0.01) (Table 1). On the other hand, the formation of ROS was significantly increased at the highest tested concentration of EGCG (*P* < 0.001 versus controls).

The rate of lipid peroxidation estimated by TBARS levels did not differ among concentrations tested.

### 3.5. Mitochondrial Membrane Potential

**Figure 3:**
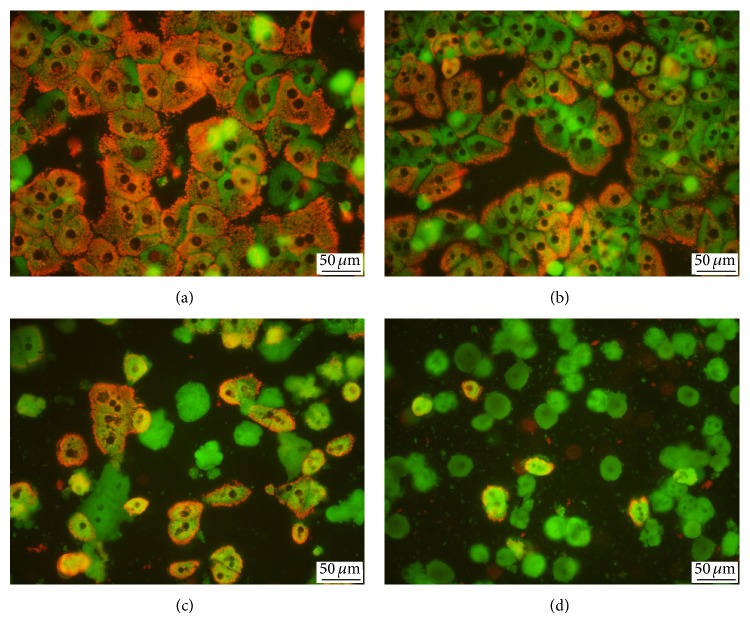
Visualization of mitochondrial membrane potential. (a) Control medium-treated hepatocytes; hepatocytes treated with EGCG at concentration of (b) 10 *μ*mol/L, (c) 30 *μ*mol/L, and (d) 100 *μ*mol/L. Note the cells with high mitochondrial membrane potential (orange) and low potential (green). JC-1 staining, objective magnification 40x.

We observed a partial loss of mitochondrial membrane potential in cells exposed to 30 *μ*mol/L EGCG for 24 h; the mitochondrial membrane potential was practically lost in hepatocytes exposed to 100 *μ*mol/L EGCG (Figures 3(a)–3(d)).

### 3.6. Mitochondrial Respiration

**Figure 4:**
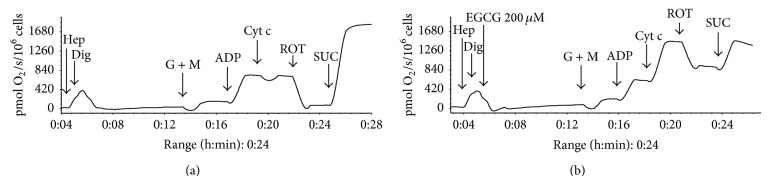
Mitochondrial respiration. Respiration of permeabilized (a) control hepatocytes and (b) hepatocytes treated with 200 *μ*mol/L EGCG for 10 minutes. Oxygen uptake is expressed as pmol oxygen/s/million cells. Abbreviations: hep, hepatocytes, dig, digitonin, G + M, glutamate + malate, ADP, adenosine diphosphate, Cyt c, cytochrome c, ROT, rotenone, and SUC, succinate.

The oxygen consumption is expressed in %, where 100% is oxygen consumption of control hepatocytes at state 4 (after addition of glutamate and malate) for given experiment. All other values are recounted to this value for given experiment. As shown in Table 2, the oxygen consumption at state 4 displayed a dose-dependent increase in cells exposed to EGCG for 10 minutes (*P* < 0.001 for 200 *μ*mol/L) giving evidence of an uncoupling of oxidative phosphorylation. State 3 (ADP-stimulated respiration of complex I) exerted only a nonsignificant trend of lowering oxygen consumption with increasing concentration of EGCG. Respiratory control ratio showed a dose-dependent decline after the exposure to EGCG for 10 minutes (*P* < 0.05 for 200 *μ*mol/L), further indicating the uncoupling of oxidative phosphorylation. Moreover, the oxygen consumption rose rapidly after the addition of exogenous cytochrome c in EGCG-treated cells (*P* < 0.05 for 200 *μ*M EGCG) suggesting disruption of the outer mitochondrial membrane. Inhibitory effect of rotenone on oxygen consumption using complex I substrates was weakened in a dose-dependent manner in cells treated with EGCG in the presence of cytochrome c (significant from 50 *μ*M concentration of EGCG); this phenomenon was not observed after addition of rotenone without previous presence of exogenous cytochrome c. Considering this effect, it was not possible to assess the influence of EGCG on complex II after addition of rotenone in the same sample (Table 2). Typical curves of respiration of permeabilized control and 200 *μ*M EGCG-treated hepatocytes are depicted in Figures 4(a) and 4(b).

## 4. Discussion

In the present study, we observed a dose-dependent toxicity of EGCG from 10 *μ*M concentration. The concentration of 5 *μ*mol/L was recognized as safe, even though it caused a significant decrease in albumin production by cultured hepatocytes.

Our results suggest toxicity at 20x lower concentration than in a study of Galati et al. [43]. The difference may have been caused by different endpoints; the other group determined the 50% lethal concentration (LD_50_), whereas we considered as toxic the minimal concentration with negative impact on cell viability. Furthermore, Galati et al. exposed the hepatocytes for maximum of 3 hours, whereas we used the 24 h interval. After 4-hour incubation, even the highest tested dose of EGCG (100 *μ*mol/L) did not exert any effect on the activity of LDH in culture medium.

We observed a decline in ROS formation after the exposure to low doses of EGCG; however, the formation was significantly increased after the exposure to 100 *μ*mol/L. Schmidt et al. [42] identified similar toxic concentrations as Galati, but they used various green tea extracts rather than EGCG in analytical grade. It was suggested that green tea extract may be less toxic due to the presence of other compounds, such as vitamin C [46]. On the other hand, the toxic concentration found in the present study is the same as in our previous study in hepatocytes isolated from rats after partial hepatectomy or laparotomy [59].

It is not very likely that the plasmatic concentration of EGCG often exceeds 10 *μ*mol/L because the oral bioavailability of EGCG is very low [60]. However, EGCG can reach approximately 4x higher concentration in the liver than in the plasma; therefore, the hepatocytes can be exposed for a short period to a concentration of 48.4 *μ*mol/kg after oral ingestion of 500 mg/kg body weight EGCG in rats [61]. In accord with these data, exposure to EGCG in a dose of 200 mg/kg induced no toxicity while the dose of 2000 mg/kg was lethal to rats [62]. Long-term exposure to 500 mg/kg EGCG was also reported as safe for the rat liver [48]. Chen et al. reported that EGCG in a dose of 75 mg/kg body weight intraperitoneally (i.p.) caused no morbidity in mice [22]. Other authors observed increased mortality in mice at 50 mg/kg EGCG i.p. [46]. The kinetics of EGCG is different in humans: a single dose of 525 mg EGCG led to a plasmatic level of 4.41 *μ*mol/L in Japanese subjects [63] and a dose of 1600 mg EGCG led to an average level of 7.4 *μ*mol/L in Caucasian subjects [64]. The concentrations would be much higher after intravenous application [60].

Our findings of the effects of EGCG on mitochondrial respiration are at variance with results of the other group, where the authors reported a negligible effect of EGCG on normal liver mitochondria [53]. However, the range of concentrations used in our study was larger. We found a dose-dependent increase in the oxygen consumption at state 4 which may indicate uncoupling of oxidative phosphorylation [65]. We also observed a nonsignificant trend of decreasing state 3 respiration by higher concentrations of EGCG (50–200 *μ*mol/L). This decline seems to be caused primarily by the loss of some cytochrome c from intermembranous space due to injury of the outer mitochondrial membrane and not by the direct inhibition of respiratory complex I by EGCG. Significant damage of the outer mitochondrial membrane was found using the highest dose of EGCG which was documented by an increase of respiration after addition of cytochrome c. Our hypothesis of the inability of EGCG to inhibit directly complex I is supported by results of Lagoa et al. [66] who found that epicatechin, another catechin of green tea, exerted no significant inhibitory effect on respiratory complex I at high concentration (100 *μ*mol/L). EGCG-induced uncoupling of oxidative phosphorylation is further supported by a dose-dependent decline of RCR.

We also found a curious effect of high doses of EGCG (50–200 *μ*mol/L) on the inhibition of respiration in the presence of complex I substrates and cytochrome c by rotenone. Under such conditions, EGCG attenuated in a dose dependent manner the inhibitory effect of rotenone on oxygen consumption. Since this action of EGCG was not observed in the absence of cytochrome c, we hypothesize that EGCG at high doses can directly reduce cytochrome c and thus provide electrons for cytochrome c oxidase. A similar effect was described in some flavonoids including epicatechin [66].

Our observations of proapoptotic action of EGCG are in accord with our previous study [59] as well as with findings of other authors [11, 52, 67] but at variance with some others [49]. In our experiment, EGCG-induced apoptosis was proved by caspase 3 activity, DAPI staining, and oligonucleosomal DNA fragmentation (data not shown). Interestingly, after exposure to 100 *μ*M EGCG, the maximum activity of caspase 3 peaked at 12 h and then declined. There was also a decrease in DAPI-positive apoptotic cells after 24 h incubation with 100 *μ*M EGCG. These data together with the highest LDH leakage and loss of mitochondrial membrane potential in this group suggest shift in the mode of cell death from apoptosis to necrosis. Rupture of the plasma membrane is a cardinal feature of necrosis that results in release of cellular constituents including LDH [68]. Moreover, the participation of necrosis was confirmed by trypan blue exclusion test showing a significant increase in trypan blue stained cells after 20 and 16 hours of cultivation with 30 and 100 *μ*M EGCG, respectively (data not shown). The relatively more pronounced increase in the activity of caspase 8 than caspase 9 suggests main contribution of the extrinsic apoptotic pathway. This may seem surprising because we observed EGCG-induced injury to the outer mitochondrial membrane with release of cytochrome c from intermembranous space which is known to trigger mitochondrial pathway of apoptosis. Possible explanation of this effect can be found in the direct action of EGCG. Since only the oxidized form of cytochrome c can induce caspase activation via the apoptosome [69] and EGCG is probably able to reduce cytochrome c, mitochondrial pathway of apoptosis in EGCG toxicity is of low importance. Similar protective effect of some other flavonoids against apoptosis was reported [66]. Chemically related substance, epigallocatechin, induced apoptosis in breast cancer cell lines, but not in normal breast epithelial cells [70].

Susceptibility to extrinsic apoptotic pathway was analyzed by El Naga et al., who reported upregulation of TRAIL death receptors in HepG2 cells after treatment with 153 *μ*mol/L EGCG [67]. However, we were not able to reproduce their findings in our previous study [59]. It is possible that the EGCG-induced death receptor upregulation is specific for cancer cell lines or occurs after very high concentrations of EGCG. The inhibition of TNF*α* production by low concentrations of EGCG observed in our study is in accord with the inhibitory effect of EGCG on TNF*α* production* in vivo* found in literature [33, 71]. In contrast to a low dose of EGCG, the higher dose (30 *μ*mol/L) elevated significantly concentration of TNF*α* in the culture medium. Considering TNF*α* as an important ligand of apoptotic death receptors, the effect of EGCG on TNF*α* production may play a substantial role in the activation of extrinsic pathway of apoptosis.

In conclusion, EGCG in a concentration of 10 *μ*mol/L displayed* in vitro* toxicity to primary rat hepatocytes. In addition, EGCG at high doses led to an uncoupling of mitochondrial oxidative phosphorylation and to damage to the outer mitochondrial membrane. We also showed that EGCG-induced apoptosis is activated mainly by the extrinsic pathway.

## Figures and Tables

**Table 1:** Biochemical parameters of cultured hepatocytes. LDH leakage (% of total LDH activity), WST-1 test (% of respective controls), albumin production (ng/mL), activity of caspases 3, 8, and 9 in cell lysate (% of respective controls), production of TBARS in cell lysate (*μ*mol/L), reactive oxygen species (ROS) formation using DCFDA (% of respective controls), and TNF*α* levels in culture medium (pg/mL). All values represent the 24-hour interval. ∗, ∗∗, and ∗∗∗ versus medium-treated controls; *P* < 0.05, 0.01, and 0.001, respectively. n/a = not measured.

	EGCG concentration (*μ*mol/L)							
	0	5	10	15	20	30	50	100
LDH leakage (*n* = 6)	16.1 ± 0.9	16.3 ± 0.6	**24.9 ± 1.7** ^***^	**37.7 ± 1.8** ^***^	**50.2 ± 1.3** ^***^	**55.3 ± 1.4** ^***^	**56.5 ± 0.8** ^***^	**66.7 ± 2.6** ^***^
WST-1 (*n* = 6)	100.0 ± 1.8	103.1 ± 2.2	**88.1 ± 3.0** ^*^	**51.2 ± 12.5** ^***^	**30.4 ± 8.2** ^***^	**10.5 ± 1.7** ^***^	**2.7 ± 0.2** ^***^	**1.5 ± 0.1** ^***^
Albumin (*n* = 6)	60.0 ± 4.4	**49.9 ± 8.9** ^**^	**38.5 ± 3.5** ^***^	**31.3 ± 3.2** ^***^	**21.7 ± 3.8** ^***^	**18.5 ± 0.9** ^***^	**11.6 ± 1.5** ^***^	**6.6 ± 1.4** ^***^
Caspase 3 (*n* = 6)	100 ± 13	121 ± 15	**690 ± 45** ^***^	**1100 ± 77** ^***^	**1212 ± 50** ^***^	**1274 ± 65** ^***^	**1491 ± 44** ^***^	**763 ± 108** ^***^
Caspase 8 (*n* = 8)	100.0 ± 18.5	n/a	108.8 ± 15.7	n/a	n/a	**339.9 ± 34.6** ^***^	**306.2 ± 32.9** ^***^	n/a
Caspase 9 (*n* = 8)	100.0 ± 12.3	n/a	105.6 ± 7.2	n/a	n/a	**116.0 ± 7.0** ^*^	**120.0 ± 11.1** ^**^	n/a
TBARS (*n* = 6)	0.49 ± 0.08	0.42 ± 0.04	0.45 ± 0.06	0.60 ± 0.23	0.47 ± 0.11	0.44 ± 0.05	0.46 ± 0.09	0.54 ± 0.09
ROS (*n* = 8)	100 ± 8.6	98 ± 8.4	**82 ± 9.5** ^**^	**80 ± 8.5** ^**^	87 ± 4.6	96 ± 12.5	110 ± 9.0	**131 ± 11.5** ^***^
TNF*α* (*n* = 5)	80 ± 8.7	19 ± 15.4	71 ± 17.4	130 ± 5.0	209 ± 32.8	**253 ± 23.1** ^*^	149 ± 21.8	99 ± 24.9

**Table 2:** EGCG-induced changes in mitochondrial respiration. The consumption of oxygen is expressed in %, where 100% is oxygen consumption of control hepatocytes at state 4 for given experiment. Other values are recounted to this value for given experiment and respiratory control ratio is calculated. G + M, glutamate + malate, ADP, adenosine diphosphate, Cyt c, cytochrome c, Rot, rotenone, SUC, succinate, and RCR I, respiratory control ratio of complex I. (*n* = 5-6) ∗, ∗∗, and ∗∗∗ versus medium-treated controls; *P* < 0.05, 0.01, and 0.001, respectively.

Substrates added	EGCG concentration (*μ*mol/L)				
	0 (control)	10	50	100	200
G + M	100	108.8 ± 8.8	115.2 ± 15.9	114.1 ± 13.7	**134.9 ± 10.3** ^***^
ADP	561.1 ± 129.4	623.9 ± 125.8	481.6 ± 48.0	506.8 ± 117.6	452.6 ± 136.4
Cyt c	561.2 ± 129.8	648.4 ± 115.0	598.8 ± 89.0	750.9 ± 158.8	**842.9 ± 189.0** ^*^
Rot	61.4 ± 14.2	76.1 ± 7.9	**177.2 ± 55.3** ^*^	**299.3 ± 72.8** ^***^	**446.0 ± 106.8** ^***^
SUC	1191.6 ± 25.4	1203.4 ± 122.8	1282.7 ± 78.8	1217.4 ± 53.7	1104.0 ± 146.7
RCR I	5.7 ± 1.1	5.7 ± 1.0	4.2 ± 0.7	4.5 ± 1.4	**3.3 ± 0.8** ^**^

## References

[1] Kim H.-S., Quon M. J., Kim J.-A. (2014). New insights into the mechanisms of polyphenols beyond antioxidant properties; lessons from the green tea polyphenol, epigallocatechin 3-gallate. *Redox Biology*.

[2] Moravcová A., Červinková Z., Kučera O., Mezera V., Lotková H. (2014). Antioxidative effect of epigallocatechin gallate against D-galactosamine-induced injury in primary culture of rat hepatocytes. *Acta Medica*.

[3] Thangapandiyan S., Miltonprabu S. (2013). Epigallocatechin gallate effectively ameliorates fluoride-induced oxidative stress and DNA damage in the liver of rats. *Canadian Journal of Physiology and Pharmacology*.

[4] Kuzu N., Bahcecioglu I. H., Dagli A. F., Ozercan I. H., Ustündag B., Sahin K. (2008). Epigallocatechin gallate attenuates experimental non-alcoholic steatohepatitis induced by high fat diet. *Journal of Gastroenterology and Hepatology*.

[5] Sahin K., Tuzcu M., Gencoglu H. (2010). Epigallocatechin-3-gallate activates Nrf2/HO-1 signaling pathway in cisplatin-induced nephrotoxicity in rats. *Life Sciences*.

[6] Thangapandiyan S., Miltonprabu S. (2014). Epigallocatechin gallate supplementation protects against renal injury induced by fluoride intoxication in rats: role of Nrf2/HO-1 signaling. *Toxicology Reports*.

[7] Akhlaghi M., Bandy B. (2012). Preconditioning and acute effects of flavonoids in protecting cardiomyocytes from oxidative cell death. *Oxidative Medicine and Cellular Longevity*.

[8] Devika P. T., Stanely Mainzen Prince P. (2008). (-)Epigallocatechin-gallate (EGCG) prevents mitochondrial damage in isoproterenol-induced cardiac toxicity in albino Wistar rats: a transmission electron microscopic and *in vitro* study. *Pharmacological Research*.

[9] Sriram N., Kalayarasan S., Sudhandiran G. (2009). Epigallocatechin-3-gallate augments antioxidant activities and inhibits inflammation during bleomycin-induced experimental pulmonary fibrosis through Nrf2-Keap1 signaling. *Pulmonary Pharmacology and Therapeutics*.

[10] Surh Y.-J., Kundu J. K., Na H.-K. (2008). Nrf2 as a master redox switch in turning on the cellular signaling involved in the induction of cytoprotective genes by some chemopreventive phytochemicals. *Planta Medica*.

[11] Lambert J. D., Elias R. J. (2010). The antioxidant and pro-oxidant activities of green tea polyphenols: a role in cancer prevention. *Archives of Biochemistry and Biophysics*.

[12] Raza H., John A. (2007). In vitro protection of reactive oxygen species-induced degradation of lipids, proteins and 2-deoxyribose by tea catechins. *Food and Chemical Toxicology*.

[13] Han S. G., Han S.-S., Toborek M., Hennig B. (2012). EGCG protects endothelial cells against PCB 126-induced inflammation through inhibition of AhR and induction of Nrf2-regulated genes. *Toxicology and Applied Pharmacology*.

[14] Lin Y.-L., Cheng C.-Y., Lin Y.-P., Lau Y.-W., Juan I.-M., Lin J.-K. (1998). effect of green tea leaves through induction of antioxidant and phase II enzymes including superoxide dismutase, catalase, and glutathione S-transferase in rats. *Journal of Agricultural and Food Chemistry*.

[15] Khan S. G., Katiyar S. K., Agarwal R., Mukhtar H. (1992). Enhancement of antioxidant and phase II enzymes by oral feeding of green tea polyphenols in drinking water to SKH-1 hairless mice: possible role in cancer chemoprevention. *Cancer Research*.

[16] Tipoe G. L., Leung T. M., Liong E. C., Lau T. Y. H., Fung M. L., Nanji A. A. (2010). Epigallocatechin-3-gallate (EGCG) reduces liver inflammation, oxidative stress and fibrosis in carbon tetrachloride (CCl_4_)-induced liver injury in mice. *Toxicology*.

[17] Yang F., Oz H. S., Barve S., de Villiers W. J. S., McClain C. J., Varilek G. W. (2001). The green tea polyphenol (-)-epigallocatechin-3-gallate blocks nuclear factor-*κ*B activation by inhibiting I*κ*B kinase activity in the intestinal epithelial cell line IEC-6. *Molecular Pharmacology*.

[18] Giakoustidis D. E., Giakoustidis A. E., Iliadis S. (2010). Attenuation of liver ischemia/reperfusion induced apoptosis by epigallocatechin-3-gallate via down-regulation of NF-*κ*B and c-Jun expression. *Journal of Surgical Research*.

[19] Byun E. H., Fujimura Y., Yamada K., Tachibana H. (2010). TLR4 signaling inhibitory pathway induced by green tea polyphenol epigallocatechin-3-gallate through 67-kDa laminin receptor. *The Journal of Immunology*.

[20] Yasuda Y., Shimizu M., Sakai H. (2009). (-)-Epigallocatechin gallate prevents carbon tetrachloride-induced rat hepatic fibrosis by inhibiting the expression of the PDGFRbeta and IGF-1R. *Chemico-Biological Interactions*.

[21] Zhen M.-C., Wang Q., Huang X.-H. (2007). Green tea polyphenol epigallocatechin-3-gallate inhibits oxidative damage and preventive effects on carbon tetrachloride-induced hepatic fibrosis. *Journal of Nutritional Biochemistry*.

[22] Chen J.-H., Tipoe G. L., Liong E. C. (2004). Green tea polyphenols prevent toxin-induced hepatotoxicity in mice by down-regulating inducible nitric oxide-derived prooxidants. *The American Journal of Clinical Nutrition*.

[23] Yumei F., Zhou Y., Zheng S., Chen A. (2006). The antifibrogenic effect of (-)-epigallocatechin gallate results from the induction of de novo synthesis of glutathione in passaged rat hepatic stellate cells. *Laboratory Investigation*.

[24] Oz H. S., Chen T. S. (2008). Green-tea polyphenols downregulate cyclooxygenase and Bcl-2 activity in acetaminophen-induced hepatotoxicity. *Digestive Diseases and Sciences*.

[25] Abe K., Ijiri M., Suzuki T., Taguchi K., Koyama Y., Isemura M. (2005). Green tea with a high catechin content suppresses inflammatory cytokine expression in the galactosamine-injured rat liver. *Biomedical Research*.

[26] Zhou J., Farah B. L., Sinha R. A. (2014). Epigallocatechin-3-Gallate (EGCG), a green tea polyphenol, stimulates hepatic autophagy and lipid clearance. *PLoS ONE*.

[27] Chung M.-Y., Park H. J., Manautou J. E., Koo S. I., Bruno R. S. (2012). Green tea extract protects against nonalcoholic steatohepatitis in ob/ob mice by decreasing oxidative and nitrative stress responses induced by proinflammatory enzymes. *The Journal of Nutritional Biochemistry*.

[28] Fiorini R. N., Donovan J. L., Rodwell D. (2005). Short-term administration of (-)-epigallocatechin gallate reduces hepatic steatosis and protects against warm hepatic ischemia/reperfusion injury in steatotic mice. *Liver Transplantation*.

[29] Skrzydlewska E., Ostrowska J., Farbiszewski R., Michalak K. (2002). Protective effect of green tea against lipid peroxidation in the rat liver, blood serum and the brain. *Phytomedicine*.

[30] Skrzydlewska E., Ostrowska J., Stankiewicz A., Farbiszewski R. (2002). Green tea as a potent antioxidant in alcohol intoxication. *Addiction Biology*.

[31] Arteel G. E., Uesugi T., Bevan L. N. (2002). Green tea extract protects against early alcohol-induced liver injury in rats. *Biological Chemistry*.

[32] Kaviarasan S., Sundarapandiyan R., Anuradha C. V. (2008). Epigallocatechin gallate, a green tea phytochemical, attenuates alcohol-induced hepatic protein and lipid damage. *Toxicology Mechanisms and Methods*.

[33] Saito Y., Mori H., Takasu C. (2014). Beneficial effects of green tea catechin on massive hepatectomy model in rats. *Journal of Gastroenterology*.

[34] Mezera V., Kučera O., Moravcová A., Peterová E., Červinková Z. (2014). Epigallocatechin gallate does not accelerate the early phase of liver regeneration after partial hepatectomy in rats. *Digestive Diseases and Sciences*.

[35] Katiyar S. K., Afaq F., Perez A., Mukhtar H. (2001). Green tea polyphenol (-)-epigallocatechin-3-gallate treatment of human skin inhibits ultraviolet radiation-induced oxidative stress. *Carcinogenesis*.

[36] Lee M. H., Kwon B.-J., Koo M.-A., You K. E., Park J.-C. (2013). Mitogenesis of vascular smooth muscle cell stimulated by platelet-derived growth factor-bb is inhibited by blocking of intracellular Signaling by epigallocatechin-3-*O*-gallate. *Oxidative Medicine and Cellular Longevity*.

[37] Potenza M. A., Marasciulo F. L., Tarquinio M. (2007). EGCG, a green tea polyphenol, improves endothelial function and insulin sensitivity, reduces blood pressure, and protects against myocardial I/R injury in SHR. *American Journal of Physiology—Endocrinology and Metabolism*.

[38] Lorenz M., Wessler S., Follmann E. (2004). A constituent of green tea, epigallocatechin-3-gallate, activates endothelial nitric oxide synthase by a phosphatidylinositol-3-OH-kinase-, cAMP-dependent protein kinase-, and Akt-dependent pathway and leads to endothelial-dependent vasorelaxation. *The Journal of Biological Chemistry*.

[39] Hirsova P., Kolouchova G., Dolezelova E. (2012). Epigallocatechin gallate enhances biliary cholesterol secretion in healthy rats and lowers plasma and liver cholesterol in ethinylestradiol-treated rats. *European Journal of Pharmacology*.

[40] Jimenez-Saenz M., Martinez-Sanchez M. d. C. (2006). Acute hepatitis associated with the use of green tea infusions. *Journal of Hepatology*.

[41] Lambert J. D., Kennett M. J., Sang S., Reuhl K. R., Ju J., Yang C. S. (2010). Hepatotoxicity of high oral dose (-)-epigallocatechin-3-gallate in mice. *Food and Chemical Toxicology*.

[42] Schmidt M., Schmitz H.-J., Baumgart A. (2005). Toxicity of green tea extracts and their constituents in rat hepatocytes in primary culture. *Food and Chemical Toxicology*.

[43] Galati G., Lin A., Sultan A. M., O'Brien P. J. (2006). Cellular and in vivo hepatotoxicity caused by green tea phenolic acids and catechins. *Free Radical Biology and Medicine*.

[44] Nakagawa H., Hasumi K., Woo J.-T., Nagai K., Wachi M. (2004). Generation of hydrogen peroxide primarily contributes to the induction of Fe(II)-dependent apoptosis in Jurkat cells by (-)-epigallocatechin gallate. *Carcinogenesis*.

[45] Pohanka M., Sobotka J., Stetina R. (2011). Sulfur mustard induced oxidative stress and its alteration by epigallocatechin gallate. *Toxicology Letters*.

[46] Goodin M. G., Bray B. J., Rosengren R. J. (2006). Sex- and strain-dependent effects of epigallocatechin gallate (EGCG) and epicatechin gallate (ECG) in the mouse. *Food and Chemical Toxicology*.

[47] Valenti D., de Rasmo D., Signorile A. (2013). Epigallocatechin-3-gallate prevents oxidative phosphorylation deficit and promotes mitochondrial biogenesis in human cells from subjects with Down's syndrome. *Biochimica et Biophysica Acta*.

[48] Meng Q., Velalar C. N., Ruan R. (2008). Regulating the age-related oxidative damage, mitochondrial integrity, and antioxidative enzyme activity in Fischer 344 rats by supplementation of the antioxidant epigallocatechin-3-gallate. *Rejuvenation Research*.

[49] Kaviarasan S., Ramamurthy N., Gunasekaran P., Varalakshmi E., Anuradha C. V. (2007). Epigallocatechin-3-gallate(-)protects Chang liver cells against ethanol-induced cytotoxicity and apoptosis. *Basic and Clinical Pharmacology and Toxicology*.

[50] Jimenez-Lopez J. M., Cederbaum A. I. (2004). Green tea polyphenol epigallocatechin-3-gallate protects HepG2 cells against CYP2E1-dependent toxicity. *Free Radical Biology and Medicine*.

[51] Zheng J., Ramirez V. D. (2000). Inhibition of mitochondrial proton F0F1-ATPase/ATP synthase by polyphenolic phytochemicals. *British Journal of Pharmacology*.

[52] Valenti D., de Bari L., Manente G. A. (2013). Negative modulation of mitochondrial oxidative phosphorylation by epigallocatechin-3 gallate leads to growth arrest and apoptosis in human malignant pleural mesothelioma cells. *Biochimica et Biophysica Acta—Molecular Basis of Disease*.

[53] Weng Z., Zhou P., Salminen W. F. (2014). Green tea epigallocatechin gallate binds to and inhibits respiratory complexes in swelling but not normal rat hepatic mitochondria. *Biochemical and Biophysical Research Communications*.

[54] Berry M. N., Edwards A. M., Barritt G. J., Burdon R. H., van Knippenberg P. H. (1991). High-yield preparation of isolated hepatocytes from rat liver. *Isolated Hepatocytes Preparation, Properties and Application*.

[55] Kučera O., Endlicher R., Roušar T. (2014). The effect of *tert*-butyl hydroperoxide-induced oxidative stress on lean and steatotic rat hepatocytes *in vitro*. *Oxidative Medicine and Cellular Longevity*.

[56] Kučera O., Al-Dury S., Lotková H., Roǔar T., Rychtrmoc D., Červinková Z. (2012). Steatotic rat hepatocytes in primary culture are more susceptible to the acute toxic effect of acetaminophen. *Physiological Research*.

[57] Ohkawa H., Ohishi N., Yagi K. (1979). Assay for lipid peroxides in animal tissues by thiobarbituric acid reaction. *Analytical Biochemistry*.

[58] Červinková Z., Křiváková P., Lábajová A. (2009). Mechanisms participating in oxidative damage of isolated rat hepatocytes. *Archives of Toxicology*.

[59] Mezera V., Kucera O., Moravcova A., Peterova E., Cervinkova Z. (2014). The effect of epigallocatechin gallate on hepatocytes isolated from normal and partially hepatectomized rats. *Canadian Journal of Physiology and Pharmacology*.

[60] Chen L., Lee M.-J., Li H., Yang C. S. (1997). Absorption, distribution, and elimination of tea polyphenols in rats. *Drug Metabolism and Disposition*.

[61] Nakagawa K., Miyazawa T. (1997). Absorption and distribution of tea catechin, (-)-epigallocatechin-3-gallate, in the rat. *Journal of Nutritional Science and Vitaminology*.

[62] Isbrucker R. A., Edwards J. A., Wolz E., Davidovich A., Bausch J. (2006). Safety studies on epigallocatechin gallate (EGCG) preparations. Part 2: dermal, acute and short-term toxicity studies. *Food and Chemical Toxicology*.

[63] Nakagawa K., Okuda S., Miyazawa T. (1997). Dose-dependent incorporation of tea catechins, (-)-epigallocatechin-3-gallate and (-)-epigallocatechin, into human plasma. *Bioscience, Biotechnology and Biochemistry*.

[64] Ullmann U., Haller J., Decourt J. P. (2003). A single ascending dose study of epigallocatechin gallate in healthy volunteers. *Journal of International Medical Research*.

[65] Bosetti F., Baracca A., Lenaz G., Solaini G. (2004). Increased state 4 mitochondrial respiration and swelling in early post-ischemic reperfusion of rat heart. *The FEBS Letters*.

[66] Lagoa R., Graziani I., Lopez-Sanchez C., Garcia-Martinez V., Gutierrez-Merino C. (2011). Complex I and cytochrome c are molecular targets of flavonoids that inhibit hydrogen peroxide production by mitochondria. *Biochimica et Biophysica Acta*.

[67] El Naga R. N. A., Azab S. S., El-Demerdash E., Shaarawy S., El-Merzabani M., Ammar E.-S. M. (2013). Sensitization of TRAIL-induced apoptosis in human hepatocellular carcinoma HepG2 cells by phytochemicals. *Life Sciences*.

[68] Gores G. J., Herman B., Lemasters J. J. (1990). Plasma membrane bleb formation and rupture: a common feature of hepatocellular injury. *Hepatology*.

[69] Brown G. C., Borutaite V. (2008). Regulation of apoptosis by the redox state of cytochrome *c*. *Biochimica et Biophysica Acta*.

[70] Vergote D., Cren-Olivé C., Chopin V. (2002). (-)-Epigallocatechin (EGC) of green tea induces apoptosis of human breast cancer cells but not of their normal counterparts. *Breast Cancer Research and Treatment*.

[71] Yang F., de Villiers W. J. S., McClain C. J., Varilek G. W. (1998). Green tea polyphenols block endotoxin-induced tumor necrosis factor- production and lethality in a murine model. *Journal of Nutrition*.

